# Polysaccharide-Polyplex Nanofilm Coatings Enhance Nanoneedle-Based Gene Delivery and Transfection Efficiency

**DOI:** 10.1002/smll.202202303

**Published:** 2022-06-30

**Authors:** Daniel Hachim, Juzhi Zhao, Jash Bhankharia, Raquel Nuñez-Toldra, Liliana Brito, Hyejeong Seong, Michele Becce, Liliang Ouyang, Christopher L. Grigsby, Stuart G. Higgins, Cesare M. Terracciano, Molly M. Stevens

**Affiliations:** Department of Materials, Imperial College London, London SW7 2AZ, UK; Department of Materials, Imperial College London, London SW7 2AZ, UK; Department of Bioengineering, Imperial College London, London SW7 2AZ, UK; National Heart and Lung Institute, Imperial College London, London SW3 6LY, UK; Department of Materials, Imperial College London, London SW7 2AZ, UK; Department of Materials, Imperial College London, London SW7 2AZ, UK; Department of Materials, Imperial College London, London SW7 2AZ, UK; Department of Materials, Imperial College London, London SW7 2AZ, UK; Department of Medical Biochemistry and Biophysics, Karolinska Institute, Stockholm 171 65, Sweden; Department of Materials, Imperial College London, London SW7 2AZ, UK; National Heart and Lung Institute, Imperial College London, London SW3 6LY, UK; Department of Materials, Imperial College London, London SW7 2AZ, UK

**Keywords:** gene delivery, nanofilms, nanoneedles, polyplexes, transfection

## Abstract

Non-viral vectors represent versatile and immunologically safer alternatives for nucleic acid delivery. Nanoneedles and high-aspect ratio nanostructures are unconventional but interesting delivery systems, in which delivery is mediated by surface interactions. Herein, nanoneedles are synergistically combined with polysaccharide-polyplex nanofilms and enhanced transfection efficiency is observed, compared to polyplexes in suspension. Different polyplex-polyelectrolyte nanofilm combinations are assessed and it is found that transfection efficiency is enhanced when using polysaccharide-based polyanions, rather than being only specific for hyaluronic acid, as suggested in earlier studies. Moreover, results show that enhanced transfection is not mediated by interactions with the CD44 receptor, previously hypothesized as a major mechanism mediating enhancement via hyaluronate. In cardiac tissue, nanoneedles are shown to increase the transfection efficiency of nanofilms compared to flat substrates; while in vitro, high transfection efficiencies are observed in nanostructures where cells present large interfacing areas with the substrate. The results of this study demonstrate that surface-mediated transfection using this system is efficient and safe, requiring amounts of nucleic acid with an order of magnitude lower than standard culture transfection. These findings expand the spectrum of possible polyelectrolyte combinations that can be used for the development of suitable non-viral vectors for exploration in further clinical trials.

## 1 Introduction

The world has witnessed decades of research and advances in nucleic acid delivery, culminating with the first generation of non-viral nucleic acid therapeutics for Coronavirus Disease (COVID-19), approved by many health agencies across the globe. Before this important landmark, a limited number of viral-based nucleic acid therapeutics have previously been approved as last treatment options for retinal dystrophy, acute lymphoblastic leukemia, and B-cell lymphoma.^[1]^ Future nucleic acid therapeutics are in development for other cancers, tissue regeneration, and treatment of different types of inherited and acquired diseases. Most current delivery approaches in the clinic rely on the use of viral vectors due to their high efficiency in delivering nucleic acids intracellularly and escaping immune surveillance mechanisms.^[2–5]^ However, the high cost of production, limited scalability, and safety concerns due to potentially adverse immunological reactions pose significant barriers toward safe clinical use and approval of nucleic acid therapeutics.^[2–5]^ For those reasons, non-viral vectors have gained more attention as inexpensive and safer alternatives for nucleic acid delivery. These typically involve negatively charged nucleic acid cargo encapsulation by cationic lipids or polycations to form liposomes and polyplexes, respectively.^[4–7]^ The primary mechanism of intracellular nucleic acid delivery of non-viral vectors occurs via endocytosis mechanisms mediated by clathrin-coated pits, phagocytosis, micropinocytosis, and membrane fusion; followed by endolysosomal escape to reach the cell nucleus.^[8–10]^ Synthetic vectors can be engineered to increase cargo loading, biodegrade, target specific cells and exhibit extended shelf-life.^[5–7,11,12]^ The main limitation of these vectors is the balance between transfection efficiency and cytotoxicity. For example, polyplexes made of poly(ethylene imine) (PEI), a widely used polycation, are highly effective but simultaneously highly cytotoxic due to the quaternary amine in the structure and lack of degradation.^[5,12–14]^ Conversely, synthetic strategies aiming to reduce the cytotoxicity of polycations have resulted in decreased transfection levels.^[5,12–14]^ Therefore, attempts to develop a non-viral delivery system with high transfection efficiency and minimal cytotoxicity are currently under investigation. An attractive polycationic candidate is poly(CBA*-co-*4-amino-1-butanol) (pABOL), which possesses a disulfide bond in the structure that renders it biodegradable by enzymatic reduction of intracellular esterases such as reduced glutathione (GSH) and thioredoxin reductases.^[15,16]^ Polyplexes comprised of pABOL with various nucleic acid payloads have sizes around a few hundred nanometers have shown no cytotoxicity at optimal concentrations to obtain the highest levels of transfection, comparable to 25 kDa branched PEI.^[15,17–19]^

Nanoneedles and other high-aspect ratio nanostructures are unconventional but interesting systems for intracellular nucleic acid delivery.^[20,21]^ There has been much debate whether these nanostructures facilitate cargo delivery into the cytosol by directly penetrating the cell membrane, or through other mechanisms.^[20–23]^ Studies have suggested that for cells seeded onto high-aspect ratio nanostructures, under the influence of gravity alone, the rate of spontaneous penetration of the membrane is relatively low,^[24]^ and is strongly influenced by the specific biochemical environment of the nanostructures and cells.^[20,25]^ Recently, it has been observed a higher proportion of clathrincoated pits and caveolae present at the cell-nanoneedle interface,^[20,26]^suggesting that endocytosis mechanisms are involved in cargo uptake. Elsewhere, the delivery effect of high-aspect ratio nanostructured surfaces can also be enhanced through the application of external stimuli, those including mechanical interfacing forces and electroporation techniques.^[20,21,27–30]^ Regardless, nanoneedles and other high-aspect ratio nanostructures have shown successful delivery of nucleic acids with minimal toxicity in vitro, including plasmid DNA (pDNA) and small interfering RNA (siRNA).^[27,29,31–33]^ In vivo, a study showed that porous nanoneedles increased angiogenesis after delivery of a vascular endothelial growth factor (VEGF) plasmid, with no evidence of toxicity or adverse reactions in the tissue.^[33]^ Another aspect to consider is the relative increase in surface area created by nano-structuring a surface. This may impact cargo uptake, in a similar fashion to high-aspect ratio nanoparticles, which have been linked to increased uptake via endocytosis.^[34,35]^ Regardless, there is limited control over the amount of nucleic acids loaded and length of release as they are merely adsorbed on the surface of the substrate.

Ultrathin coatings, created using layer-by-layer (LbL) self-assembled nanofilms, are highly versatile systems for nucleic acid delivery. The layer assembly of these coatings is typically mediated by the electrostatic interactions of two or more polyelectrolytes, which are deposited in alternating cycles onto the surface of a charged substrate.^[36–38]^ The multilayered nature of these coatings allows exquisite control over loading, release kinetics, and porosity (from smooth to highly porous surfaces); while their nanometer-thickness scale preserves the architecture of substrates with complex geometries.^[39,40]^ This has been used to deliver a variety of cargoes that include proteins,^[39–42]^drugs,^[43,44]^nucleic acids,^[45–48]^ and particle systems.^[49,50]^ Therefore, we have combined self-assembled polyplex-polysaccharide nanofilms with high-aspect ratio nanostructures to synergistically enhance nucleic acid delivery and transfection efficiency. We studied the influence of distinct polyplex-coated nanoneedle architectures and other high-aspect ratio structures on transfection efficiency. We also assessed various polyplex-polyelectrolyte combinations, and we found that polysaccharide-based polyanions enhanced the transfection efficiency of pABOL polyplexes, while other polyanions proved to be detrimental to delivery. Interestingly, a few studies have previously claimed that hyaluronate/hyaluronic acid (HA), an anionic polysaccharide and non-sulfated glycosaminoglycan (GAG), resulted in enhanced nucleic acid delivery via liposomes, nanoparticles, and PEI-polyplexes, showing good levels of transfection.^[47,51–58]^ These studies suggested that the presence of HA in these vectors could enhance nucleic acid delivery by CD44-mediated uptake, where HA binds its receptor CD44, followed by internalization and intracellular release of the vector.^[52–54]^ A second proposed mechanism is via structural properties of the polymer, where HA would modulate the electrostatic interactions within the polyplex and/or establish hydrogen bonding with nucleic acids, which would facilitate the release from the complex.^[51,53]^ Interestingly, our studied nanofilm combinations showed that enhanced plasmid delivery was not specifically linked to HA, but rather to polysaccharide-based polyanions. Moreover, results showed that these effects were not mediated by interactions with the CD44 receptor. Systematic studies were performed to clarify the role of polysaccharide-based polyanions with enhanced transfection efficiency, expanding the spectrum of possible polyelectrolyte combinations that can be used to develop non-viral vectors with high delivery efficiency and low cytotoxicity.

## 2 Results and Discussion

### 2.1 Plasmid DNA-Polyplex Characterization

**Figure 1:**
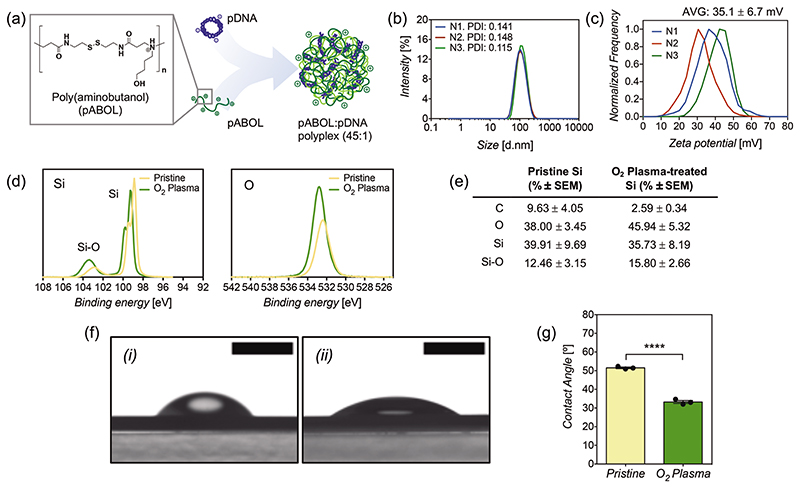
a) Schematics of polyplex formation between pDNA and pABOL in a mass ratio of 1:45. b) Particle diameter of polyplexes via dynamic light scattering (DLS) and c) zeta potential of polyplexes. Three samples shown (*N* = 3) correspond to the mean of three measurements. d) Silicon and oxygen spectra using X-ray photoelectron spectroscopy (XPS) on the surface of pristine silicon nanoneedles and O_2_ plasma-treated silicon nanoneedles. e) XPS surface composition of pristine silicon and O_2_ plasma-treated silicon nanoneedles. Results are the mean of three samples (*N* = 3) ± SEM. f) Representative images of contact angle measurements on i) pristine silicon nanoneedles and ii) O_2_ plasma-treated silicon nanoneedles. Scale bars represent 2 mm. g) Contact angle values as the mean of three replicates (*N* = 3) ± SEM. Statistical significance difference as (****) *p* < 0.0001, using a two-tailed *t*-test.

Polyplexes loaded into the coating were made of a bio-reducible polycationic polymer, poly(cystamine bisacrylamide*-co-*4-amino-1-butanol), hereafter referred to as pABOL, in which its backbone structure contains a disulfide bond that is broken down intracellularly after endocytosis via esterases, releasing the nucleic acid cargoes contained within the polyplex complex.^[15,16]^ Polyplexes were loaded with a pCAG-GFP, a plasmid coding for green fluorescent protein (GFP), by mixing them in a mass ratio of 45:1 (Figure 1a), which corresponds to an N/P ratio of 38, which is the ratio of positively charged amino groups in pABOL to the number of negatively charged phosphate groups in the pDNA. This optimized mass ratio was chosen from previous studies using pABOL polyplexes for delivery of nucleic acids.^[17–19]^ This method results in polyplexes of 104 ± 3.6 nm in average size, with good polydispersity (PDI: 0.135); and a surface zeta potential of +35.1 ± 6.7 mV (Figure 1b,c), making them positively charged, which facilitates electrostatic deposition and loading onto the coating.

### 2.2 Polyplex-Polysaccharide Coated Nanoneedle Characterization

An optimal surface charge of the silicon nanoneedles is a key requirement to allow LbL deposition of the first polycationic chitosan layer. The surface of silicon nanoneedles (50 nm tip, 4.5 μm height) was cleaned from any remaining impurities using isopropanol:acetone (1:1) followed by oxygen plasma treatment. X-ray photoelectron spectroscopy (XPS) revealed that the plasma treatment increased the proportion of silicon oxide (103 eV) over pure silicon (98–100 eV doublet) (Figure 1d,e). Silicon oxide is known to provide negatively charged oxide ions.^[59,60]^ The increase in surface oxidation is also further supported by an increase in oxygen (533 eV) (Figure 1d,e). Contact angle measurements showed that the hydrophilicity of the nanoneedle surface significantly increased after plasma treatment (Figure 1f,g), which corresponds with the increased SiO_2_ seen under XPS.

**Figure 2:**
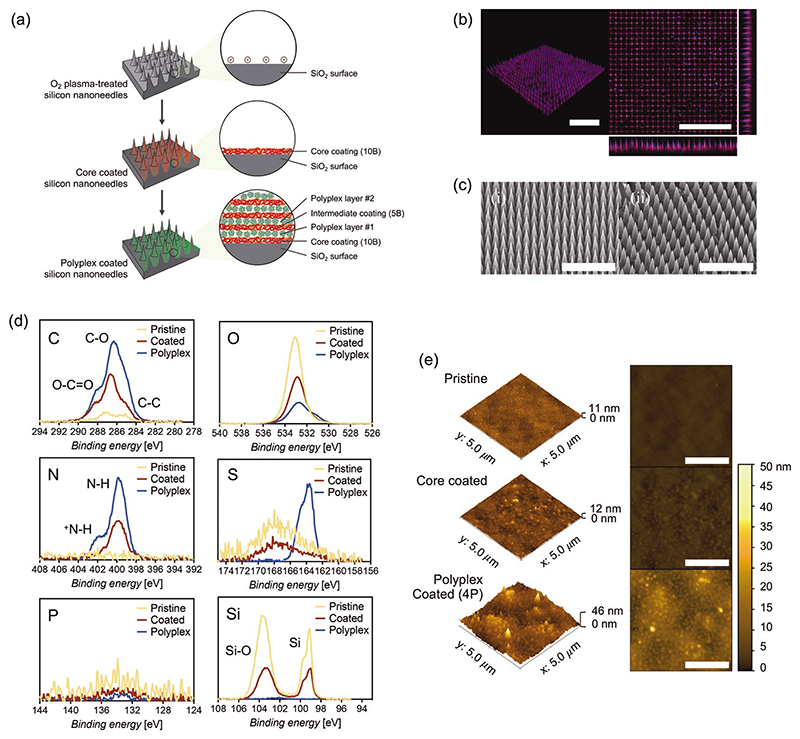
a) Polysaccharide-polyplex nanofilm coating procedure of silicon nanoneedles and high-aspect ratio nanostructures. b) Confocal microscopy representative image of silicon nanoneedles coated with hyaluronate-chitosan-pDNA polyplex nanofilms. The nanofilm was stained with TAMRA (red) and pDNA-polyplexes were stained with DAPI (blue). Scale bars represent 20 μm. Separate images for each channel can be found in Figure S1, Supporting Information. c) Scanning electron microscopy (SEM) images of i) pristine nanoneedles and ii) polyplex-nanofilm coated nanoneedles. Scale bars represent 10 μm. d) Carbon, oxygen, nitrogen, sulfur, phosphorus, and silicon spectra obtained via XPS from pristine, coated (no polyplex) and polyplex-coated (4 layers) nanoneedles. e) Surface topography characterization via atomic force microscopy (AFM) for pristine, coated (no polyplex) and polyplex coated (4 layers) flat silicon substrates. Scale bars represent 2 μm.

After plasma treatment, nanoneedles were coated using a LbL procedure via cycles of self-assembled deposition of chitosan (polycation) and HA (polyanion), mediated by opposite electrostatic interactions. We used a core coating of 10 bilayers (10B) as a supporting surface for further polyplex loading, which has been shown to ensure a uniform coating with no gaps present.^[39,40]^ The first polyplex layer, made of net positively charged complexes, was deposited onto the last negatively charged HA layer of the core coating, and additional polyplex layers were loaded within an intermediate coating of 5 bilayers (5B) (Figure 2a). The presence and uniformity of the nanofilm were evaluated via confocal microscopy, using tetramethylrhodamine (TAMRA) to stain the coating (reacting to amino groups in chitosan) and DAPI to stain pDNA-polyplexes. Confocal images showed that the entire nanoneedle surface is coated and polyplexes are distributed in the coating (Figure 2b; Figure S1, Supporting Information). Differences in fluorescence intensity and missing nanoneedles are most likely a consequence of needle fracture during handling or artifacts during nanoneedle fabrication. Another important note is that the architecture of nanoneedles is preserved after the coating procedure, corroborated via SEM imaging (Figure 2c), which is essential to study the specific effects of high-aspect ratio nanostructures on transfection efficiency. XPS has been used to provide a more exhaustive confirmation of the components present in the coating (Figure 2d). The predominant presence of C-O (286 eV) and O-C=O (288 eV) structures show the polysaccharide structures from hyaluronic acid and chitosan, while the specific presence of nitrogen peaks (400 and 402 eV) confirms the presence of chitosan. The presence of pABOL is confirmed explicitly by a peak in sulfur at 164 eV, but also an increased ratio in protonated nitrogen (^+^ N-H) species at 402 eV due to the tertiary amine from the polymer. Due to the comparatively low amount of DNA in the polyplexes (1:45 pDNA:pABOL), further diluted into the coating, the phosphorus signal from pDNA is low but still detected. We have further evaluated the surface composition with increasing numbers of polyplex and seen that the presence of silicon species diminished until complete disappearance by 4 polyplex layers (Figure S2, Supporting Information). This indicates how the coating increases thickness with more layers until the x-ray beam is not able to penetrate deep enough to detect the underlying silicon substrate. At this particular point, the surface composition and atomic percentages seen under XPS resemble more accurately the real elemental proportion of species in the coating. Since atomic force microscopy (AFM) cannot readily be used on high-aspect ratio structures like nanoneedles, the surface roughness of the coating was assessed on flat silicon substrates. After coating, the surface roughness increased compared to the relatively smooth surface of pristine silicon (Figure 2e). It is possible to observe spheric-like shapes of ≈100 nm in diameter on the surface of polyplex-coated flat silicon surfaces, which may correspond to the polyplexes, which are distributed uniformly throughout the surface, and embedded within the coating. This increase in surface roughness translates into an increased surface area, expected to enhance the interactions at the interface between cells and nanoneedles and hence cell uptake of polyplexes.

### 2.3 Release and Degradation of Polyplex-Polysaccharide Nanofilms

**Figure 3:**
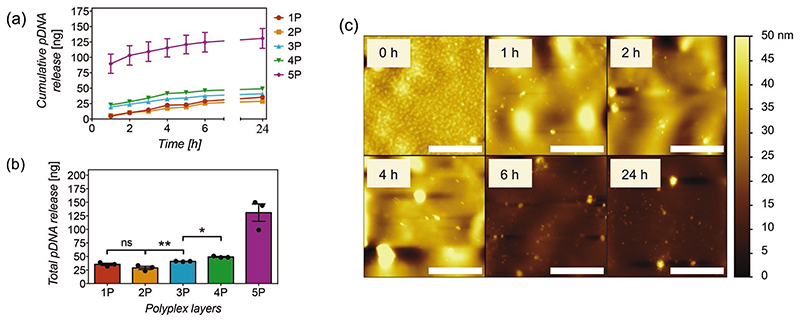
a) Cumulative release of pCAG-GFP plasmid DNA (nanograms) versus time (hours) and b) total amount of pCAG-GFP released at 24 h from 8 × 8 mm silicon substrates coated with hyaluronate-chitosan nanofilms containing 1 to 5 layers of polyplexes (1P to 5P) in PBS buffer pH 7.4 containing 10 mM of reduced glutathione at 37 °C. Points and bars represent the mean of three replicates (*N* = 3) ± SEM. Statistical significance as (*) *p* < 0.05 and (**) *p* < 0.01, using one-way ANOVA with Sidak’s test to compare 1P to 4P. (ns) non-significant difference. 5P was not considered in the analysis. Linear trend post-test with *p* < 0.0001. c) Nanofilm degradation over time (hours) in function of surface topography via AFM assessments. Scale bars represent 2 μm.

To study, quantify and optimize the loading and intracellular delivery of pDNA of the nanofilms as a function of polyplex layers, flat silicon substrates of known surface area (8 × 8 mm^2^) coated with polyplex nanofilms were assayed in a release buffer containing a physiological intracellular concentration of reduced glutathione (10 mM),^[61]^ and the pDNA was quantified from aliquots taken at different time points using a PicoGreen assay. Release kinetics analyses showed similar trends for most samples regardless of the number of polyplex layers. Nanofilms with 3P and 4P released ≈40% of their pDNA loaded within the first hour, reaching complete release by 6 h, as evidenced by a plateau in the release curves (Figure 3a). Nanofilms with 1P and 2P released ≈20%of their total content by 1 h, also reaching completion by 6 h. As expected, the amount of pDNA loaded in the coating increased with the number of polyplex layers, all of them on the nanogram scale (Figure 3b). While the growth of material deposition in LbL films has been described to occur exponentially via dipping methods,^[62,63]^ there was an unexpected and sudden increase in the amount of pDNA in the 5-polyplex layers sample, accompanied by a high variability. AFM assessments on these samples showed abnormal deposition and the presence of material aggregation in the nanofilms (Figure S3, Supporting Information), which are not present in 4-polyplex layer nanofilms. Increased washing times or changes in polymer concentration did not change these outcomes, suggesting they occur unavoidably. These anomalies are likely a major contributor to the aberrant loading and release kinetics, hence not considered for statistical analysis.

The detection of polyplexes and the clear differences in surface roughness between coated and non-coated silicon substrates, allow feasible tracking of degradation via AFM over time using the same experimental conditions used for release assays. The results are consistent with release assays. The degradation of polyplexes occurs mainly within the first hour, while the presence of coating material seems to remain in the substrate for at least 4 h, then entirely degraded at 6 h, with surface roughness quite comparable to pristine controls (Figure 3c).

### 2.4 Transfection Efficiency of Polyplex-Polysaccharide Nanofilms

**Figure 4:**
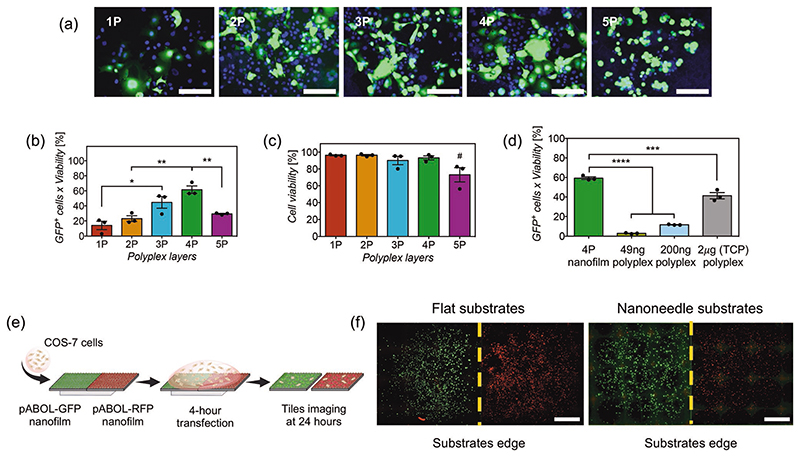
a) Fluorescent microscopy images of COS-7 cells transfected and expressing pCAG-GFP plasmid (green) using silicon substrates coated with nanofilms containing 1 to 5 polyplex layers (1P to 5P). DAPI (blue) was used as a nuclear counterstain. Scale bars represent 100 μm. b) Quantification of transfection efficiency and c) viability via flow cytometry analyses of COS-7 cells transfected via silicon substrates coated with nanofilms containing 1 to 5 polyplex layers (1P to 5P) after 24 h incubation. Bars represent the mean ± SEM (*N* = 3). Statistical significance as (*) *p* < 0.05 and (**) *p* <0.01, using one-way ANOVA and Sidak’s test. (#) statistical significance with *p* < 0.05, using one-way ANOVA with Sidak’s test to compare 5P to all other groups. (ns) non-significant difference. d) Transfection efficiency of COS-7 cells seeded on silicon substrates coated with 4-polyplex layers versus polyplexes in suspension containing 49 and 200 ng of pDNA on top of silicon substrates; and a standard culture transfection of polyplexes using 2 μg of pCAG-GFP in TCP. Bars represent the mean ± SEM (*N* = 3). Statistical significance as (****) *p* < 0.0001, using one-way ANOVA and Sidak’s test. e) Schematics of the procedure to assess surface-mediated transfection using two contiguous silicon substrates on COS-7 cells, one containing pCAG-GFP (green, left) and the other pCAG-RFP (red, right). f) Tiled and stitched fluorescent microscopy images from the whole surface of two contiguous flat and nanoneedle substrates, expressing GFP (green) or RFP (red). Scale bars represent 2 mm.

Transfection efficiency on COS-7 cells was studied as a function of the number of polyplex layers in the nanofilms to observe whether there is a correlation with the amount of released plasmid. The transfection efficiency, which is the percentage of GFP expression adjusted to cell viability, was evaluated 24-h post-transfection via fluorescence microscopy and flow cytometry. Results showed an increasing trend in transfection efficiency with the first four polyplex layers (Figure 4a,b), which correlated to an increasing amount of released pDNA. However, the 5-polyplex layers coated nanoneedles were significantly less effective in transfection than the 4-polyplex layers, even though they released more than twice the amount of pDNA. Also, 5-polyplex layers resulted in lower viability (Figure 4c). This is likely a consequence of the abnormal nanofilm deposition observed under AFM and aberrant release previously observed. Regardless, 4-polyplex layer coatings resulted in the highest transfection efficiency and were therefore used for all further studies. An optimal number of intermediate chitosanhyaluronic acid bilayers between polyplexes was also assessed to maximize transfection efficiency. We found that each polyplex layer, contained within five intermediate bilayers, resulted in the highest and most reproducible transfection levels (Figure S4, Supporting Information). No additional bilayers were assessed as this would make the procedure impractical, hence all studies have been done with five intermediate bilayers.

We investigated whether the HA-chitosan-polyplex nanofilm enhances plasmid delivery and transfection efficiency compared to polyplexes alone. The transfection efficiency of silicon substrates coated with 4-polyplex layer nanofilms was compared to polyplexes in suspension containing 49 ng of plasmid, equivalent amount of pDNA released on 4-polyplex nanofilms (Figure 3b), but also compared to polyplexes containing 200 ng of pDNA, which is equivalent to standard concentrations used with polyplexes (2 μg/500 μL). COS-7 cells were seeded on flat silicon to maintain consistent experimental conditions. Results with polyplexes in suspension containing 49 ng of plasmid resulted in minimal transfection efficiency, compared to 4-polyplex layer nanofilm substrates with a transfection efficiency of 59.3% (Figure 4d). Polyplexes containing 200 ng of plasmid increased transfection efficiency to 11.5%, but still significantly lower than 4-polyplex nanofilms. Transfection was also performed in standard culture conditions, with COS-7 cells already adhered to tissue culture plastic (TCP) wells, using polyplexes in suspension containing 2 μg of plasmid in 500 μL, which resulted in transfection efficiency of 41.3%. This shows that adhesion of cells before transfection is important to obtain higher transfection efficiency using polyplexes in suspension, while nanofilms do not present this limitation. Therefore, these results demonstrate consistently that HA-chitosan nanofilms enhance the transfection efficiency of polyplexes, using an amount of plasmid an order of magnitude lower than standard transfection with polyplexes in culture, but also most non-viral commercial vectors, including liposomes and nanoparticles. In fact, an order of magnitude higher in the amount of plasmid in the polyplexes (2 μg) was not enough to equal the transfection efficiency obtained with nanofilms. This matches previous findings, in which HA has been incorporated during polyplex formation and shown to enhance nucleic acid delivery.^[47,51–58]^ Studies to evaluate whether the specific presence of HA is required for an enhanced transfection have been performed and are discussed later.

Intracellular delivery of cargoes by high-aspect ratio structures occurs at the cell-material interface. This highly localized delivery is a consequence of cargoes being physisorbed to the surface and not readily released to the surrounding environment.^[20–23]^ On the other hand, delivery and release of molecules in LbL coatings rely on the degradation properties of the polymers forming the coating and its architecture. For instance, the release of protein cargoes triggered by enzymatic action in the host tissue has been shown to affect the biological response of cells ≈50 μm from the tissue material interface.^[39]^ Alternatively, LbL films containing intracellular nucleic acid cargoes have been shown to depend more on cell uptake and delivery only occurs in cells directly interfacing the surface of the coated substrates.^[45–48]^ To assess whether polyplexes remain within the nanofilms for highly localized surface-mediated transfection or diffuse away from the surface during transfection, we have used two contiguous coated silicon substrates: one loaded with pCAG-GFP (coding for GFP); and the second one containing pCAG-RFP, coding for red fluorescent protein (RFP), placed on top of a PDMS sheet to ensure tight adhesion within a tissue culture well-plate, as shown in Figure 4e. Transfection was done by adding a single-cell suspension to cover both substrates simultaneously, allowing for possible polyplex diffusion, and incubated together for 4 h. After 24 h post-transfection, results showed COS-7 cells expressing either GFP or RFP on their corresponding sides of the coated substrates, with a clear separation of transfected cells in the contiguous edge of the substrates (Figure 4f). Therefore, polyplexes remained confined to each side of the substrate. No apparent diffusion or release of polyplexes was observed, confirming a surface-mediated mechanism of transfection, which means that cells or tissues must interface the coating for transfection to occur. The immobilization and possible concentration of polyplexes at the surface of the material may contribute to the observed enhanced transfection efficiency, with an order of magnitude lower in the amount of plasmid compared to polyplexes in suspension. This also demonstrates the potential of nanofilms for spatial patterning of transfection and generation of gradients in multicellular systems and tissue regeneration, respectively.

### 2.5 Influence of High-Aspect Ratio Nanostructures on Transfection Efficiency

**Figure 5:**
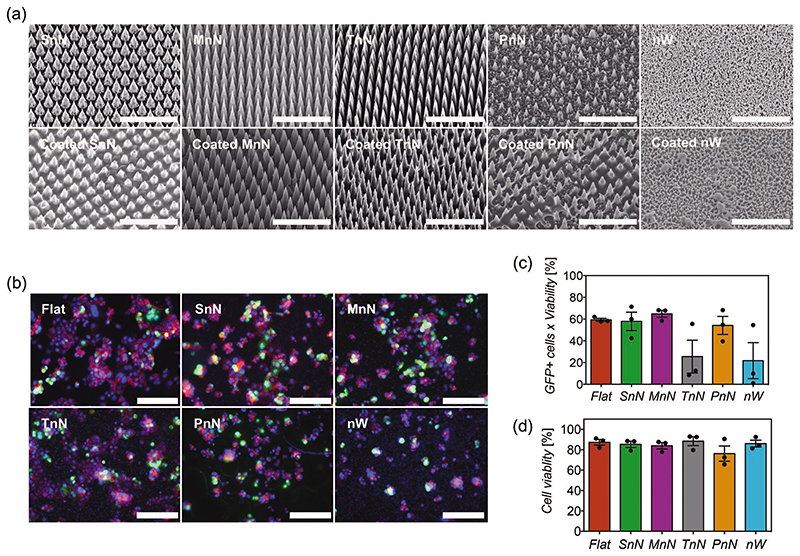
a) SEM images of pristine high-aspect ratio nanostructures (top) and nanostructures coated with 4-polyplex nanofilms (bottom). SnN, MnN, TnN, and PnN correspond to small, medium, tall, and porous nanoneedles, respectively. nW corresponds to nanowires. Scale bars represent 10μm. b) Fluorescent microscopy images of COS-7 cells transfected on distinct high-aspect ratio nanostructures coated with 4-polyplex nanofilms. GFP^+^ cells are shown in green, while the cytoskeleton of cells has been stained with phalloidin (red) and cell nuclei counterstained with DAPI (blue). Scale bars represent 100 μm. c) Quantification of transfection efficiency and d) viability via flow cytometry analyses of COS-7 cells transfected with different coated high-aspect ratio nanostructures. Bars represent the mean ± SEM (*N* = 3). Statistical significance was found using one-way ANOVA (*p* < 0.05), but significances between specific means were not found with Sidak’s post-test.

Considering that an optimal tissue or cell interface with the coated substrate is essential for an efficient transfection of genes, the surface area would be an important parameter for both the coating and high-aspect ratio nanostructures. Hence we have studied transfection efficiency with nanostructures of increasing surface area and several architectures. In increasing order of relative surface area, we compared transfection efficiency of COS-7 cells on flat silicon, short solid nanoneedles (SnN, height 3 μm), medium solid nanoneedles (MnN, height 4.5 μm), tall solid nanoneedles (TnN, height 7 μm), porous nanoneedles (PnN, height 5.5 μm) and nanowires (nW, height 400 nm). SEM was used to demonstrate that the architecture of these high-aspect ratio nanostructures was preserved after the coating procedure, ensuring that the differences in transfection efficiency are attributed to the nanostructures and not to other artifacts caused by the coating. As shown in Figure 5a, the architecture of all solid nanoneedles was preserved. The overall shape of porous nanoneedles was mainly preserved, however, some features in the nanoneedles seemed to be too small to be preserved and were coated during the procedure. The dimensions of the nanowires were small, but the coating was able to maintain the overall architecture of their features, with the presence of some aggregates.

In this study, transfection efficiency was complemented with phalloidin staining to assess differences in cell morphology in response to each nanostructure. Results revealed similar transfection efficiencies for flat silicon, small nN, medium nN, and porous nN, while tall nanoneedles and nanowires show a lower average in transfection efficiency and inconsistent results with higher variance between repeats (Figure 5b,c). Considering these findings and their corresponding cell morphological staining, these inconsistencies in transfection for both TnN and nW substrates seem to be associated with poor interfacing area between cells and nanostructures. As previously mentioned, an optimal cell-surface interface is essential for transfection, but it seems that an increased surface area in the substrate does not necessarily translate to a larger area of cell contact. Instead, in this case, the architecture of high-aspect ratio structures seems to be more important, including height, spacing, or porosity. For subsequent experiments, we used medium nanoneedles, which have the lowest variability in transfection efficiency of all solid nanoneedles and better preserved high-aspect ratio structure after coating than porous needles. Medium nanoneedles have also been chosen over flat substrates as we have seen evidence that nanoneedles aid tissue delivery in a number of different contexts,^[21,33,64,65]^ but also in our ex vivo studies, as it will be shown in a later section.

### 2.6 Role of Polyanion Structure on Enhanced Transfection Efficiency

As previously mentioned, it has been recently hypothesized that HA enhances nucleic acid delivery.^[47,51–58]^ The proposed mechanisms have linked this enhancement to (1) the chemical structure of HA – modulating the electrostatic interactions in the polyplex via hydrogen bonding,^[51,53]^ and (2) CD44-mediated cell uptake of the complex via interactions with HA.^[52–54]^ We first studied the role of the chemical structure of HA on transfection efficiency, comparing coatings made of different polyanions in combination with chitosan. The presence of all polyelectrolytes and pDNA-polyplexes in these coatings was confirmed successfully via XPS (Figure S5, Supporting Information).

**Figure 6:**
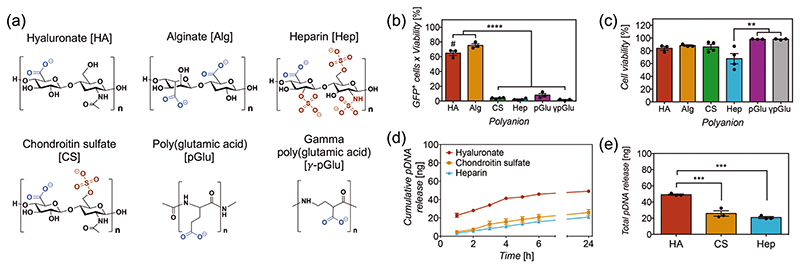
a) Chemical structures of the polyanions assessed in the study, with their anionic groups highlighted in a different color. b) Transfection efficiency and c) viability of COS-7 cells transfected using nanoneedles with polyplex nanofilms containing distinct polyanions. Bars represent the mean ± SEM (*N* = 3–4). Statistical significance as (**) *p* < 0.01 and (****) *p* < 0.0001, using one-way ANOVA and Sidak’s test. (#) *p* < 0.05 compared to alginate. d) Cumulative release of pCAG-GFP plasmid DNA (nanograms) versus time (hours) and e) total amount of pCAG-GFP released at 24 h, from silicon substrates coated with 4-polyplex nanofilms containing hyaluronate (HA), chondroitin sulfate (CS) and heparin (Hep); in PBS buffer pH 7.4 containing 10 mM of reduced glutathione at 37 °C. Points and bars represent the mean of three replicates (*N* = 3) ± SEM. Statistical significance as (***) *p* < 0.001, using one-way ANOVA and Sidak’s test.

HA is composed of D-glucuronic acid and N-acetyl-D-glucosamine disaccharide units, and it is the only non-sulfated member of the GAG family that plays important roles in the extracellular matrix and regulation of cellular responses.^[66–68]^ Therefore, transfection efficiency was tested in other members of the same family such as chondroitin-4-sulfate (CS), a GAG with low sulfation degree, in which constituent disaccharide units are D-glucuronic acid and N-acetyl-D-galactosamine.^[68,69]^ Similarly to HA, CS has also been shown to bind CD44.^[70,71]^ Heparin (Hep), a GAG with a high sulfation degree, has also been tested. This is composed of sulfated uronic acid and sulfated D-glucosamine.^[68,72]^ Hep has been shown to bind an exon v3 variant of CD44 to regulate the activity of specific growth factors.^[71,73]^ The chemical structures of all three GAGs and all studied polyanions are shown in Figure 6a. Transfection with both sulfated GAGs resulted in a drastically reduced efficiency (Figure 6b), which indicates that sulfation in the polysaccharide structure is detrimental for transfection, as discussed below. Alginate (Alg) was then chosen due to similar polysaccharide chemical structure and charge density to HA, but formed by D-mannuronate and L-guluronate sugars.^[74]^ Results revealed higher transfection efficiency than HA (75.3% versus 64.8%), demonstrating that enhanced transfection is not specific to HA (Figure 6b). The role of the polysaccharide backbone in transfection efficiency from both HA and Alg has been assessed by testing coatings with polyglutamic acid (pGlu), a polyaminoacid with the same carboxylate anion but no polysaccharide backbone. Interestingly, results showed poor transfection (8%), suggesting that a polysaccharide backbone in the polyanion would be essential to facilitate transfection.

We looked further into the detrimental effect of sulfation on transfection efficiency and evaluated whether the coating is affecting the release of pDNA, as a consequence of the high anionic charge of sulfonates in the polyanion. Release assays with both chondroitin sulfate (CS) and Hep showed lower released amounts of pDNA released, while similar release kinetics were observed (Figure 6d,e). No linear dependence between the degree of sulfation and transfection efficiency was observed. These results show that sulfation in the polyanion structure affects the release of pDNA and hence transfection efficiency. Supporting evidence indicates that sulfated polysaccharides disrupt the electrostatic interactions within polyplexes and liposomes. For example, buffers containing dextran sulfate or Hep have been used to unpack nucleic acid cargoes to quantify release.^[55,58,75,76]^ It is then possible that part of the pDNA is released from the polyplexes during the coating procedure. However, in other systems, polyplexes coated with CS have been shown to enhance transfection efficiency,^[77,78]^ which suggests that these effects may also be dependent on the architecture of the delivery system, polymer composition, and concentration. However, these differences in release amount do not account for the dramatic decrease in transfection efficiency observed, with 4.4% for CS and 1% for Hep (Figure 6B).

Polyglutamic acid promotes poor transfection compared to both HA and Alg, in which the most evident structural difference is the presence of a polysaccharide backbone. The abundant hydroxyl groups in the polysaccharide backbone serve as both hydrogen bond acceptors and donors, while pGlu predominantly has hydrogen bond acceptors and a hydrophobic segment. Previous studies using polysaccharides and other synthetic polymers for particle decoration have claimed that hydrogen bonding modifies the strength and density of electrostatic interactions between the nucleic acids and polycations, facilitating the release of cargoes from the polyplex.^[12,51,53]^ Alternatively, the higher charge density of polyglutamic acid could disrupt polyplexes as a consequence of the shorter spacing among amino acid units in polyglutamic acid compared to the sugar units in the polysaccharide backbone of both HA and Alg. To test this hypothesis, the transfection efficiency was assessed using gamma polyglutamic acid (γ-pGlu), in which glutamic acid is polymerized in the *γ* position, increasing the distance between carboxylate anions from three to five atoms which decreases the charge density to similar levels to polysaccharides: Alg with five atoms and HA with 10 atoms (Figure 6a). The results showed that the transfection efficiency using *γ*-pGlu is low, with similar efficiency to pGlu (Figure 6b), suggesting that the enhancement in transfection efficiency observed in polysaccharides is not dictated by charge density in the polyanion. The poor performance of *γ*-pGlu on delivery and transfection efficiency seems to be specific to the nanofilm-polyplex system, considering that *γ*-pGlu has shown to improve cell-uptake and transfection efficiency when incorporated in the fabrication of polyplexes.^[79–83]^

### 2.7 Role of CD44-Mediated Cell Uptake on Enhanced Transfection Efficiency

**Figure 7:**
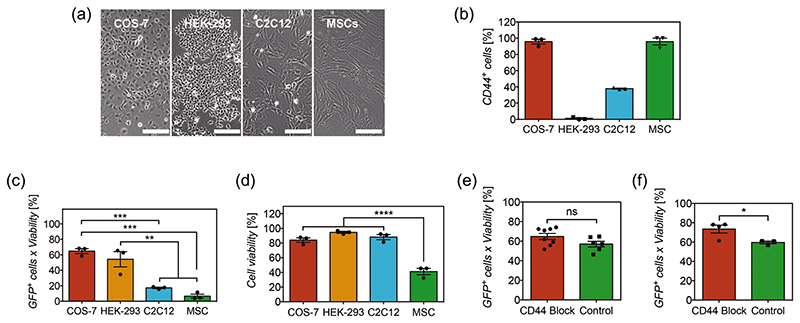
a) Images of cell types studied and b) their level of CD44 expression receptor. Scale bars represent 200 μm. Bars represent the mean ± SEM (*N* = 3). c) Transfection efficiency and d) viability of distinct cell types transfected with nanoneedles coated with 4-polyplex nanofilms. Bars represent the mean ± SEM (*N* = 3). Statistical significance as (**) *p* < 0.01, (***) *p* < 0.001 and (****) *p* < 0.0001, using one-way ANOVA and Sidak’s test. e) Transfection efficiency of COS-7 cells before and after blocking of receptor CD44, using hyaluronate-chitosan-polyplex coated nanoneedles and f) alginate-chitosan-polyplex coated nanoneedles. Bars represent the mean ± SEM (*N* = 3–8). Statistical significance as (*) *p* < 0.05, using a two-tailed *t*-test. (ns) non-statistical difference.

To test the hypothesis of CD44-mediated cell uptake, we have evaluated the transfection efficiency in cell types with and without distinct expression of CD44 (Figure 7a). We have quantified the levels of CD44 expression using antibody labeling and flow cytometry analyses (Figure 7b). COS-7 cells, a fibroblast cell line from monkey kidney,^[84]^ present high expression levels of CD44 (96%). HEK-293, an epithelial cell line from human kidney embryo^[85]^ exhibits almost no expression of CD44 (1%). C2C12, a mouse myoblast cell line,^[86]^ showed a relatively fair expression of CD44 (37.5%). Human mesenchymal stem cells (MSCs), primary cells derived from bone marrow, as expected by definition,^[87]^ exhibited a high (95.8%) expression of CD44. Transfection results have shown that the high transfection efficiency of COS-7 is correlated to the high expression of CD44 (Figure 7c). However, HEK-293T showed a similar high transfection efficiency to COS-7 cells, regardless of the minimal expression of CD44. C2C12 cells exhibited lower transfection efficiency than both COS-7 and HEK-293T cells. Surprisingly, MSCs showed marginal transfection efficiency (3%), which should have been significantly higher due to the highest expression of CD44 if CD44-mediated uptake were indeed the predominant mechanism at play.

Despite the fact that the transfection efficiency does not seem to comply with the CD44-mediated transfection hypothesis given the uncorrelated levels of CD44 expression in most of these cell types, it could be argued that HEK-293T cells could still efficiently uptake polyplexes via conventional endocytosis, or that MSCs are widely known to be inherently difficult to transfect.^[88,89]^ For further confirmation, transfection efficiency was assessed in COS-7 cells after blocking the CD44 receptor using an antibody, and compared to normal transfection. This has been done in nanoneedles coated with either HA or Alg, both with high transfection efficiency and polysaccharide-based chemical structures (Figure 6b). Blocking the CD44 receptor in COS-7 cells did not disrupt transfection efficiency. In both cases, results did not show decreased transfection efficiency after blocking CD44 receptors in COS-7 cells and unexpectedly, transfection efficiency in nanofilms containing Alg was higher after blocking (Figure 7e,f). Therefore, the results in this study consistently confirm that facilitated transfection of HA and Alg does not predominantly occur via CD44-mediated cell uptake. Instead, it relies on the polysaccharide physicochemical properties such as hydrogen bonding to facilitate intracellular delivery of the nucleic cargo.

Despite producing some results at odds with the previously-proposed CD44-mediated cell uptake mechanism, our results do confirm that HA facilitates transfection.^[47,51–58]^ We also demonstrate that transfection is not only facilitated by HA but also by additional polysaccharide-based polyanions such as Alg. We also have to consider experimental differences with prior studies where HA has likely been integrated into the bulk and surface during the formation of polyplexes, liposomes, and particles; in contrast, our procedure would likely integrate HA onto the surface of the polyplex. Discrepancies may also arise from the molecular weight of hyaluronic acid used, cell types studied and CD44 antibodies used for blocking.

### 2.8 Surface-Mediated Transfection on an Ex Vivo Cardiac Slice Model

**Figure 8:**
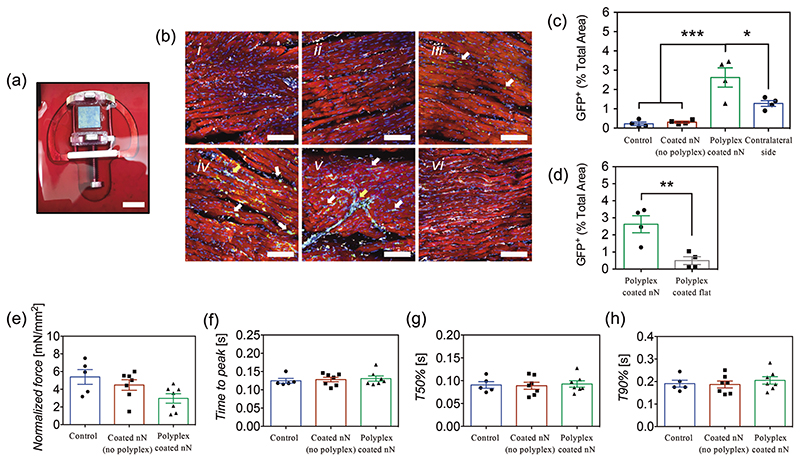
a) Polyplex-coated nanoneedle substrate interfacing cardiac slice on custom-made holders. Scale bar represents 8 mm. b) Confocal microscopy images of cardiac slices at 24 h of transfection and immunolabeled with anti-GFP (green), anti-cTNT (red), anti-vimentin (white), and DAPI (blue). Representative images correspond to i) control no chip, ii) coated (no polyplex) nanoneedles, iii) polyplex-coated flat substrates, iv,v) polyplex-coated nanoneedles, and vi) the non-interfacing side of a slice with polyplex-coated nanoneedles. Bars represent 100 μm. Separate images showing green and blue channels are shown in Figure S7, Supporting Information. The level of GFP expression in these groups was quantified via image analysis, c) as percentage of total area, d) while a comparison between GFP expression on nanoneedles and flat substrates coated with 4P-polyplex nanofilms was performed separately. Bars represent the mean ± SEM (*N* = 4). Statistical significance as (*) *p* < 0.05, (**) *p* < 0.01 and (***) *p* < 0.001, using two-tailed *t*-tests to compare two groups or one-way ANOVA with Sidak’s test for multiple groups. Traces from force transducer analyses showing e) contraction force in mN mm^−2^, f) time to peak, g) time to 50% decay, and h) time to 90% decay. Bars represent the mean ± SEM (*N* = 5–7). Differences were not statistically significant: e) *p* = 0.052, f) *p* = 0.837, g) *p* = 0.930, and h) *p* = 0.684, using one-way ANOVA (*p* < 0.05).

We evaluated the efficacy of the polyplex coated nanoneedles to transfect tissues on an ex vivo cardiac slice platform. Due to the architecture of nanoneedles and the surface-mediated delivery of the coating, transfection would occur in the outermost layer of cells in the tissue, in which in vivo imaging techniques and histological methods are limited. Cardiac slices, on the other hand, are an ideal ex vivo platform to study surface-mediated transfection on tissues, with many imaging techniques and quantification methods available.^[90,91]^ These slices were prepared from left ventricles of rats in 300 μm sections, an optimal thickness to allow nutrient and oxygen supply for prolonged survival.^[91–93]^ Polyplex coated nanoneedles or controls were interfaced for 1 h on top of the cardiac slices, fixed to a holder inside of a custom chamber filled with sufficient culture media to be in contact with the bottom side of the slice, allowing the nanoneedle substrate to adhere to the tissue before filling the chamber completely with media (Figure 8a). Similar to our in vitro studies, slices were evaluated after 24 h of incubation, during which time the coated nanoneedles stayed in contact with the tissue. The tissue viability was assessed using a Live/ Dead staining. The functionality of the slices after transfection was evaluated via contractility trace analysis from force transducer measurements, then fixed for staining.

To assess tissue transfection in the slice, a co-immunolabeling was performed using antibodies against GFP (green, transfected cells), cardiac troponin (cTNT, red, cardiomyocyte marker), vimentin (white, a stromal cell marker to label fibroblast and endothelial cells) and DAPI (blue) as counterstaining. Image analysis was performed to quantify the expression of GFP, as percentage of the total area in the imaged field. After 24 h of transfection, fibroblasts and endothelial cells showed to be transfected, as evidenced by high colocalization of GFP expression and vimentin, mainly localized between cardiomyocyte bundles and in the lining of blood vessels (Figure 8b-iv,v). Only a few discrete transfected cardiomyocytes were observed. No transfection was observed in controls groups and coated (no polyplex) nanoneedle groups (Figure 8b-i,ii). This selective transfection might be explained by the intrinsic physiological roles of these cells within the cardiac tissue, resulting in distinct cell uptake activity. For example, previous studies have shown that cardiomyocytes exhibit lower cell uptake of extracellular vesicles (EVs) compared to endothelial cells and fibroblasts,^[94,95]^ which was hypothesized to be related to the increased ability of endothelial cells and fibroblasts to receive signals from cardiomyocytes via EVs, especially under injury, process that remains to be elucidated.^[95–97]^ The observed differences in transfection among groups were correlated with quantification of GFP expression—nanoneedles with 4P-polyplex nanofilms showed 2.62% area of GFP per field, while nanofilm controls and no chip controls showed an area of 0.31% and 0.22%, respectively (Figure 8c). These results are in agreement with levels of transfection observed in COS-7 (fibroblasts), HEK-293 (epithelial cells), and C2C12 (myoblasts or muscle cells) in vitro (Figure 7), while noting that a direct comparison among these cell lines with cells in the tissue is not intended due to their metabolic and physiological differences. These results also support the highly localized surface-mediated mechanism of delivery, as transfection was only observed on the top side of the cardiac slice, where the tissue was interfacing the polyplex coated nanoneedles. In contrast, limited transfection and a lower area of GFP expression were observed on the contralateral surface (Figure 8b-vi,c). In our in vitro experiments, medium nanoneedles were selected over flat nanofilm substrates as hypothesized the penetration of nanoneedles into the tissue would increase surface interaction and transfection efficiency compared to flat. Cardiac slices were transfected with 4-polyplex layer flat nanofilms and results showed that the expression of GFP within the tissue was considerably lower than nanoneedles (Figure 8b-iii,d). Therefore, polyplex coated nanoneedles are capable of transfecting tissues with an exquisite level of localization, also ensuring a proper interface with tissues for facilitated transfection.

Cell viability assessments showed preserved viability of the cardiac tissue, with no differences in the expression of calcein and number of dead cells among slices treated with polyplex coated needles, coated nanoneedles (no polyplex), and nonanoneedle controls (Figure S6, Supporting Information). Similarly, force transducer measurements of contractility in the cardiac slices were performed to demonstrate that transfection did not alter the functionality of the tissue. Trace analysis of cardiac contractility showed preserved function of cardiac slices, with no differences in contracting force (Figure 8e), time to peak (Figure 8f), and time to decay (Figure 8g,h) observed among groups. Therefore, transfection via nanoneedles coated with polyplex-nanofilms preserves both the viability and functionality of the tissue.

In clinical settings, our surface-mediated transfection system is then advantageous in accessible tissues, where highly localized transfection and confined penetration depth is sought. For example, the epicardium is the outermost layer covering the heart, harboring a population of progenitor cells capable of regeneration in the heart, mainly dormant after birth, which makes the epicardium a highly attractive target for transfection to reactivate gene pathways that stimulate epithelial-to-mesenchymal transition.^[98,99]^ Given the highly confined layer architecture of the epicardium, a surface-mediated transfection via the polyplex-nanofilm system would hence be highly suitable for localized and efficient transfection. Transdermal delivery via nanoneedle systems may pose limitations, compared to their microscale counterparts. This route has always been an attractive and suitable target for microneedle-mediated delivery, in which many cargoes (e.g., nucleic acids, proteins, and drugs) have been delivered for therapeutic applications that include melanoma, wound healing, as well as sustained systemic delivery of drugs.^[100–102]^ In this context, the combination of nanofilms with microneedles would be well suited for skin interfacing. The limited penetration depth of nanoneedles (a few microns) would be insufficient to reach tissue below the stratum corneum, but may still be appropriate for wound healing, where the stratum corneum is compromised. Similarly, transfection or delivery of cargoes using coated nanoneedles could be performed directly on the surface of small tumors or any other tissues, as long as profound penetration is not required. All these applications would benefit from a clinical perspective by transitioning toward flexible and biodegradable high-aspect ratio nanostructures, as these would interface non-planar tissues more appropriately and allows for implantation into tissues without the need for resection surgeries. Regardless, the fabrication method of nanofilms is highly versatile and hence can be easily combined with a wide variety of delivery systems and medical devices for the delivery of therapeutic cargoes.

## 3 Conclusions

The polyplex-polysaccharide coating based on LbL assembly of nanofilms is a suitable technology to provide uniform nanometerscale coatings that preserve the architecture of nanoneedles and other high-aspect ratio nanostructures. The multilayered nature of the coating provides fine control over release kinetics and amount of pDNA loaded, which was directly correlated to transfection efficiency, with maximum efficiency at 4-polyplex layers in this particular case. A proper interface between cells and nanostructures is essential for the surface-mediated delivery of the coating, as evidenced by the highly localized delivery of polyplexes with different cargoes. High-aspect ratio nanostructures of increasing surface area were not necessarily correlated to increased cell contact, but nanostructures with poor contact with cells resulted in inconsistent transfection efficiency. While in vitro, high-aspect ratio nanostructures and flat substrates with similar cell contact exhibited comparable transfection efficiency, nanoneedles coated with polyplex nanofilms showed significantly higher transfection efficiency in tissues, compared to nanofilms in flat substrates, which is likely due to increased penetration into the tissue.

The presence of polysaccharides in the coating enhanced transfection efficiency compared to polyplexes alone, with an amount of plasmid an order of magnitude lower than standard culture transfection. Our results show that enhanced delivery is not restricted to HA as claimed in previous studies but is also seen in polysaccharide-based polyanions such as Alg. However, the use of sulfated anionic polysaccharides is detrimental, as they disrupt polyplexes and nucleic acid release. Moreover, the predominant mechanism leading to this enhancement does not occur via CD44-mediated cell uptake. Instead, the essential role of polysaccharides facilitating delivery and transfection seems to involve hydrogen bonding regulation of the electrostatic interactions with nucleic acids within the polyplexes. In ex vivo cardiac slices, transfection is observed mainly in fibroblast and endothelial cells, while cardiomyocytes do not seem to be consistently transfected, which is in agreement with in vitro studies. Transfection was predominantly observed on the side of the slice interfacing polyplex-coated nanoneedles. Viability and functional assessments show that tissue viability and function after nanoneedle transfection remain unchanged. Therefore, gene transfection in tissues via nanoneedles coated with polyplex nanofilms is biocompatible, efficient, and highly localized. This is potentially an attractive approach for clinical settings where surgical procedures enable direct tissue-nanoneedle interfacing. While further testing and trials are required, the system has potential as an off-the-shelf type device that can be maintained dry-frozen before use. Beyond nanoneedles and high-aspect ratio nanostructures, the use of this coating technology in combination with microneedles and other devices opens an entire spectrum of different clinical approaches for nucleic acid delivery with precise spatial control.

## 4 Experimental Section

### Materials

HA, chondroitin sulfate A, Hep, chitosan, Alg, and pGlu were purchased from Sigma Aldrich, UK. HPLC-grade water, isopropanol, and acetone were purchased from VWR, Germany. γ-pGlu was purchased from Carbosynth Limited, UK. Cell culture materials, anti-human/monkey CD44, anti-vimentin antibody, phalloidin, secondary antibodies, and DAPI were purchased from Thermo Fisher Scientific, UK. Anti-mouse CD44 was purchased from BD Biosciences. pCAG-GFP and pCAG-dsRFP were obtained from Addgene (MA, USA) and developed by Cepko C, et al.^[103]^ Fluc-pcDNA3 was obtained from Addgene (MA, USA) and developed by Safran M, et al.^[104]^ pABOL was obtained in-house and synthesized as previously described.^[15,16]^ P-type doped silicon wafers with 0.01 Ω cm resistivity were obtained from University Wafers, USA. Anti-GFP and anti-cardiac troponin T were obtained from Abcam, UK.

### Fabrication of High-Aspect Ratio Nanostructures

Silicon nanoneedle and nanowire patterns were generated using reactive ion etching (RIE) and photolithography as previously reported.^[33,105,106]^ Briefly, a 1200 Å layer of low-stress silicon nitride was deposited using low-pressure chemical vapor deposition (Scottish Microelectronic Centre, The University of Edinburgh, UK). Dot arrays with varying diameters and spacings were transferred to a hard mask via photolithography, using a MA6 mask aligner (Suss Microtech, Germany), NR9-250P photoresist, and RD6 developer (Futurrex, USA). RIE was done on silicon wafers using 50 sccm of CF_3_ gas, 5 sccm of O_2_ gas, 55 mTorr of pressure, and 140 W of power for 150 s. The dot-patterned wafer was mounted on a 6 inch-diameter carrier wafer using a Crystalbond 555 adhesive stick for DRIE using a deep reactive ion etcher (Surface Technology Systems, UK). Each DRIE cycle consisted of i) 130 sccm of SF_6_ gas and 6 sccm of O_2_ gas with a process pressure of 15 mTorr and power of 800 W for an 8 s etch phase and ii) 85 sccm of C_4_F_8_ gas with a process pressure of 14 mTorr and power of 600 W for a deposition phase of 6.5 s. To produce structures of 3–7 μm height, between 30 and 45 cycles were conducted. To fabricate porous nanoneedles, the patterned substrate was cleaned in 10% HF, then coated with Ag using 1 mM AgNO_3_ in 10% HF for 2 min and washed in water and isopropanol. Porosity was obtained using metal-assisted chemical etching (MACE) in 10% HF, 122 mM H_2_O_2_ for 8 min 30 s. Sharpening of the structures was done via RIE using SF_6_ plasma for 3 min 45 s at a pressure of 100 mTorr, with gas flux of 20 sccm and forward plasma bias power of 200 W. To fabricate nanowires, the native silicon oxide layer was removed using 2.83 M HF, then immediately immersed into a 2.83 M HF and 0.02 M AgNO_3_ for an electroless deposition of silver during 1 min. The reaction was stopped with sequential washes of water, isopropanol, and N_2_ drying. MACE was done with 2.83 M HF and 0.081 M H_2_O_2_ for 2 min and then stopped. Residual silver was removed by immersing the substrates into a solution of gold etchant (Sigma Aldrich, USA), for 10 min, then washed sequentially as described above. The processed wafers were then diced into 8 × 8 mm squares for further use.

### Polyplex Fabrication and Characterization

pCAG-GFP plasmid and pABOL were mixed in a mass ratio of 1:45 in 20 mM HEPES, 5% w/v D-glucose, pH 7.4, ratio optimized in previous studies.^[17–19]^ For each polyplex layer, 2 μg of pCAG-GFP was diluted in 15 μL of buffer and 90 μg of pABOL were diluted in 55 μL of buffer, then mixed and vortexed for 30 s. This procedure was also used to fabricate polyplexes containing either pCAG-dsRFP or Fluc-pcDNA3. Polyplex size and zeta potential were assessed via dynamic light scattering using a Zetasizer Nano ZSZEN3600 (Malvern Instruments, Worcestershire, UK). To do so, polyplexes in triplicate were diluted to 1 mL with HPLC-grade water, measured three times each at 25 °C.

### Fabrication of Self-Assembled Polyplex-Polysaccharide Nanofilms

To provide a more hydrophilic and negatively charged surface, patterned silicon substrates were treated with oxygen plasma, using oxygen gas at 1 mBar steady-state pressure, 100 W for 5 min in a PlasmaPrep5 instrument (Gala Instrumente GmBH). An automated layer by layer coating was done using an in-house customized 3D printer (GeeTech M2 Creator 2, China). The LbL coating was done by dipping the patterned substrates in 2 mg mL^−1^ of chitosan in 0.5% acetic acid for 10 min and three washes in HPLC-grade water, 1 min each. This was followed by dipping the substrates into a 1 mg mL^−1^ polyanion solution in water (HA, CS, Hep, Alg, pGlu, or *γ*-pGlu) for 10 min and three washes in HPLC-grade water, 1 min each. This cycle was repeated ten times to obtain a 10-bilayer core coating. To deposit a monolayer of DNA polyplexes onto the coated nanopatterns, 70 μL of a freshly prepared solution of polyplexes was placed on top of the substrate and incubated for 1 h at room temperature (RT), followed by three washes in HPLC-grade water. Additional chitosan and polyanion monolayers were deposited for a total of 5 bilayers (including polyplex), before adding the following pDNA polyplex layer. This was performed in cycles to obtain nanopatterned substrates coated with 1 to 5 polyplex layers. Core coated substrates were stored at 4 °C, while substrates containing DNA polyplexes were stored at –20 °C before use.

### Characterization of Polyplex-Polysaccharide Nanofilms

The presence and uniformity of the coating were observed via confocal microscopy, using TAMRA to stain the amino groups in the coating and DAPI to stain the DNA polyplexes. The presence of each polymer (elemental coating surface composition) in the coating was assessed via XPS on a Thermo-Fisher K Alpha XPS system (Waltham, MA, USA). To acquire the overall elemental composition, a survey of 2 scans was performed with a constant analyzer energy of 200 eV, dwell time of 25 ms, 0.5 eV step size, and an X-ray spot size of 400 μm. Single element spectra were obtained using a constant analyzer energy of 20 eV, dwell time of 50 ms, 0.1 eV step size, an X-ray spot size of 400 μm, 10 scans for silicon/ carbon/oxygen, and 20 scans for nitrogen/sulfur. Carbon peak was used as reference, with a value of 284 eV for adventitious carbon. Spectra data were analyzed using Avantage software V5.9925, (Thermo Scientific, Waltham, MA, USA). The preservation of the patterned nanostructures after coating was corroborated via scanning electron microscopy (SEM). To do so, samples were mounted and sputtered with a 10 nm layer of chromium (Q150, Quorum) and imaged using a LEO Gemini 1525 FEGSEM (Zeiss, Germany) with an accelerating voltage of 5 keV. Surface roughness and presence of polyplexes in the coating were assessed via AFM, using an Agilent 5500 AFM system (Agilent Technologies, USA), equipped with silicon nitride cantilevers (MikroMasch AFM Tips, Germany) in intermittent contact mode. Gwyddion V2.49 software was utilized for data processing.

### Degradation and Release Studies

These assays used 1 to 5 polyplex-coated flat silicon substrates of 8 × 8 mm, immersed into 500 μL of a PBS pH 7.4 buffer containing 10 mM of reduced glutathione at 37 °C. Degradation of polyplexes (4P layers) and the coating was assessed through changes in surface topography and porosity via AFM, using an Agilent 5500 (Agilent Technologies, USA). Topographical images were recorded in dry flat silicon substrates every 1 h using silicon nitride cantilevers (MikroMasch AFM Tips, Germany) in intermittent contact mode. Gwyddion V2.49 software was utilized for data processing. For release assays, aliquots were taken every hour and replaced with fresh glutathione buffered solution. Aliquots were stored at −20 °C until quantification. Released pCAG-GFP in these aliquots was quantified using PicoGreen assay, following kit instructions.

### Cell Transfection

COS-7 and HEK-293T were cultured in DMEM High Glucose (4.5 g L^−1^) +Glutamax medium supplemented with 10% v/v FBS and 1% v/v Penicillin-Streptomycin (PS), at 37 °C and 5% v/v CO_2_. C2C12 were cultured in DMEM High Glucose (4.5 g L^−1^) +Glutamax medium supplemented with 10% FBS and 1% v/v PS, at 37 °C and 5% CO_2_. Mesenchymal stem cells (MSCs) were cultured in MesenPRO RS Medium with 2% v/v Mesen-Pro Growth Supplement, 1% w/v L-glutamine and 1% v/v PS, at 37 °C and 5% CO_2_.

Cells in plates with 80–90% confluency were used for surface-mediated transfection, detached using 0.25% w/v Trypsin-EDTA, followed by two washes in Opti-MEM medium with 1% v/v Penicillin-Streptomycin. Cells were counted and resuspended in a concentration of 6 × 10^5^ cells mL^−1^ in Opti-MEM medium + 1% v/v PS. pCAG-GFP polyplex coated substrates of 8 × 8 mm were placed inside of 24-well plates and 3 × 10^4^ cells (50 μL) were placed on the top of the substrates, covering the whole coated surface of the patterned nanostructures. Cells were incubated for 4 h at 37 °C and 5% CO_2_ to allow cell adhesion and transfection, and then 500 μL of the standard medium was added to fill the well. After 24 h of incubation at 37 °C and 5% CO_2_, GFP^+^ cells were imaged under fluorescence microscopy or detached for quantification via flow cytometry. Comparisons with polyplexes alone were done by adding polyplexes (containing 49 ng and 200 ng of pCAG-GFP, maintaining same mass ratio) during cell seeding on top of silicon substrates to keep consistency, while a standard polyplex transfection was done on a 48-well TCP plate, using polyplexes (2 μg of pCAG-GFP) suspended in 500 μL of Opti-MEM medium + 1% v/v PS and added to COS-7 cells seeded 24 h in advance.

Fluorescent imaging was done in an Olympus IX71 microscope (Olympus Life Sciences, UK), cells were washed three times with PBS, fixed in 4% PFA (w/v) for 15 min on ice, and permeabilized with 0.5% (v/v) Triton-X100 for 10 min at RT, then washed three times with PBS. The cytoskeleton of cells (actin) was stained with rhodamine-labeled phalloidin (1:2000) in PBS for 1 h at RT, followed by three PBS washes. Nuclei counterstaining was done with DAPI (1:1000) in PBS, 15 min at RT before imaging. Images were analyzed with ImageJ V1.51 (NIH, USA). For flow cytometry analyses, cells in the substrates were washed with PBS and detached with 0.25% v/v Trypsin-EDTA. Cells were resuspended in 250 μL of PBS buffer containing 1 mM EDTA, 25 mM HEPES, and 1% FBS (flow cytometry buffer), then 15 μL of To-Pro-3 Iodide (ThermoFisher, UK) were added before runs to evaluate cell viability. Each test was done with 10^4^ events, using 488/530 nm laser for GFP and 640/670 nm laser for To-Pro-3 Iodide. Data were acquired on a LSR Fortessa Cell Analyser (BD BioSciences, UK) and analyzed using Flowjo V7.0.

### Surface-Mediated Transfection Assessment

Localized surface-mediated transfection was assessed using two 8 × 8 mm flat or nanoneedle (4.5 μm height) substrates: one loaded with 4-polyplex layers of pCAG-GFP (green) and another one coated with 4-polyplex layers of pCAG-RFP (red), using the same coating procedure described previously. Both nanoneedle substrates were put next to each other on top of a PDMS sheet that keeps the substrates fixed inside of a 6-well plate. Transfection was done as described above, adding 6 × 10^4^ COS-7 cells (100 μL) on the top of the two substrates, hence an 8 × 16 mm area. After 4 h of incubation, substrates were individually put in 24-well plates filled with DMEM + Glutamax medium, 10% v/v FBS, 1% v/v PS, and incubated at 37 °C and 5% CO_2_ for 24 h. Transfected cells with either GFP or RFP were imaged on the whole surface of both chips using tiling and stitching on an Axio Observer.Z1 inverted widefield microscope (Zeiss, Germany).

### CD44 Expression and Blocking

A CD44 monoclonal antibody (Thermo Scientific, UK) was used to determine the expression of CD44 in COS-7, HEK-293, C2C12, and MSCs, as well as block the receptor during transfection. Cells were detached and washed in flow cytometry buffer. 1 × 10^5^ cells in 300 μL of buffer were incubated with primary CD44 antibody (1:50) at 4 °C for 30 min. Cells were centrifuged at 300 g and washed with buffer twice, followed by incubation with secondary Alexa Fluor 555 antibody (1:250) at 4 °C for 30 min. Cells were washed three times with flow cytometry buffer before measurements. Similarly, cells were blocked with a solution of CD44 antibody (1:50) at 4 °C for 30 min. Control groups were incubated in PBS without antibody under the same conditions. After blocking, cells were washed twice with Opti-MEM and 3 × 10^4^ cells were seeded on coated nanoneedle substrates as previously described.

### Coated Nanoneedle-Based Transfection on Cardiac Slices

Cardiac slices were prepared from Sprague–Dawley male rats (300–350 g) as previously described.^[91]^ All animal procedures were performed under license by the UK Home Office, in agreement with the United Kingdom Animals (Scientific Procedures) Act 1986 and guidelines established by the European Directive on the protection of animals used for scientific purposes (2010/63/EU). The heart and the surrounding tissues of the animal were excised and immersed in cold Tyrode’s solution (pH 7.4), containing 1000 IU mL^−1^ of Hep. The left ventricle was isolated from the rest of the heart and extra-heart tissues, then opened with an incision down the interventricular septum and flattened via incisions to the papillary muscles. The tissue was mounted on a 2.5 cm^2^ specimen-holder coated in 4% agarose, epicardial side down, using surgical glue (Histoacryl Octyl Micro, Braun Surgical S.A, Catalonia). The specimen holder was placed in a vibratome bath filled with cold Tyrode’s solution, bubbled with filtered 100% O_2_. Using a high precision vibratome (7000 amz-2, Campden Instruments) with a ceramic blade, the tissue was sliced (300 μm thickness) longitudinal to the fiber orientation from the endocardium down. About four to six slices were obtained per heart. Once the slice was obtained, fiber alignment was visualized under light microscopy to cut an aligned squared slice. Custom-made plastic 3D printed rectangular holders were attached perpendicular to the fibers along the width of the slice using surgical glue. The slice was placed on custom-made stainless-steel stretchers and stretched at physiological load (17.5% stretch, equivalent to 2.2 μm sarcomere length). All length measurements were taken with calipers. Stretched slices were then placed in groups of four in custom-made culture chambers and super fused with 60 mL of oxygenated media. The culture media (Medium-199) with Earl’s salts was prepared by adding 0.1% v/v ITS (insulin-transferrin-selenium) +3% v/v PS. Hormones (4 nM adrenaline, 4 nM noradrenaline, 100 nM dexamethasone, and 2.15 nM triiodothyronine (T3)) with the addition of 20 μg mL^−1^ ascorbic acid were also added to the media to maintain the physiological properties of the slices during culture.

Polyplex coated nanoneedle chips substrates and controls (8 × 8 mm) were interfaced with the upper side of the cardiac slice for 1 h, while the media was filled to the level of the slice, to prevent substrates from floating and allow tissue adhesion. After 1 h of incubation at 37 °C and 5% CO_2_, the chamber was filled with media and cultured for 24 h under electrical field stimulation, using carbon electrodes at 1 Hz, 10 ms pulse width, and 15 V. After transfection, slices were mounted on a force transducer system (Harvard Apparatus) to assess viability and function, under field stimulation at 1 Hz, 10 ms pulse and 15 V. Cardiac muscle contraction traces generated (milli newtons versus time) were analyzed using pClamp V11 software (Molecular Devices, CA) to obtain normalized contraction force (mN mm^−2^, normalized to slice area), time to peak (s), time to decay 50% and 90% (s). Slices were then fixed with 4% w/v PFA for 15 min and rinsed in PBS for immunolabeling. Slices were blocked and permeabilized for 3 h RT with a PBS buffer containing 5% w/v BSA (Bovine Serum Albumin), 10% v/v FBS, 5% v/v Horse Serum and 1.5% v/v Triton X-100. Blocking buffer was removed and replaced with 1:10 blocking buffer:PBS containing the following primary antibodies: rabbit anti-GFP (1:1000), mouse anti-cTNT (1:500), and chicken anti-vimentin (1:3000). Slices were incubated overnight with primary antibodies at 4 °C. After incubation, slices were washed three times with PBS (30 min each) and put in a PBS buffer containing 1% w/v BSA + 0.3% v/v Triton X-100 and the following secondary antibodies: donkey anti-rabbit Alexa Fluor 488 (1:2000), donkey anti-mouse Alexa Fluor 594 (1:500), goat anti-chicken Alexa Fluor 647 (1:2000). Secondary antibodies were incubated at RT for 2 h, then washed three times with PBS (30 min each). A DAPI solution in PBS (1:1000) was added and incubated for 15 min at RT and washed three times (2 min each). For viability testing, a Live/dead staining was done following kit instructions, using nanoneedles coated with polyplexes containing Fluc-pcDNA3, to eliminate the presence of green fluorescence due to transfection. Slices were imaged using a Zeiss Axiovert Confocal Microscope (Zeiss, Germany). Image analysis to quantify area percentage and dead cells was performed in ImageJ V1.51 (NIH, USA).

### Statistical Analyses

Comparisons of means were performed using a two-tailed *t*-test or one-way analysis of variance (ANOVA), using at least *p* < 0.05 as statistical significance criteria, followed by Post Hoc tests to perform multiple comparisons. These tests were performed on GraphPad Prism V6 (California, USA). Shapiro–Wilk was used to test normality, using SPSS V28 (IBM, USA).

## Supplementary Material

Supplementary Information

## Data Availability

The data that support the findings of this study are available from the corresponding author upon reasonable request.
