# Supplementary material for: Polysaccharide-Polyplex Nanofilm Coatings Enhance Nanoneedle-Based Gene Delivery and Transfection Efficiency
Source: Small. Author manuscript; Available in PMC 2024 Jan 11. (PMC7615482; doi:10.1002/smll.202202303)
Supplement: Supplementary Information [file EMS175902-supplement-Supplementary_Information.pdf]

NANO small MICRO

Supporting Information

for *Small,* DOI: 10.1002/smll.202202303

Polysaccharide-Polyplex Nanofilm Coatings Enhance Nanoneedle-Based Gene Delivery and Transfection Efficiency

*Daniel Hachim, Juzhi Zhao, Jash Bhankharia, Raquel Nuñez-Toldra, Liliana Brito, Hyejeong Seong, Michele Becce, Liliang Ouyang, Christopher L. Grigsby, Stuart G. Higgins, Cesare M. Terracciano, and Molly M. Stevens**

Supplementary Information

Polysaccharide-polyplex nanofilm coatings enhance nanoneedle-based gene delivery and transfection efficiency.

Daniel Hachim[1,2,3], Juzhi Zhao[1,2,3], Jash Bhankharia[2], Raquel Nuñez-Toldra[4], Liliana Brito[1,2,3,4], Hyejeong Seong[1,2,3], Michele Becce[1,2,3], Liliang Ouyang[1,2,3], Christopher L. Grigsby[5], Stuart G. Higgins[1,2,3], Cesare M. Terracciano[4] and Molly M. Stevens[1,2,3,5].*

1 Department of Materials, Imperial College London, London, UK.

2 Department of Bioengineering, Imperial College London, London, UK.

3 Institute of Biomedical Engineering, Imperial College London, London, UK.

4 National Heart and Lung Institute, Imperial College London, UK.

5 Department of Medical Biochemistry and Biophysics, Karolinska Institute, Stockholm, Sweden.

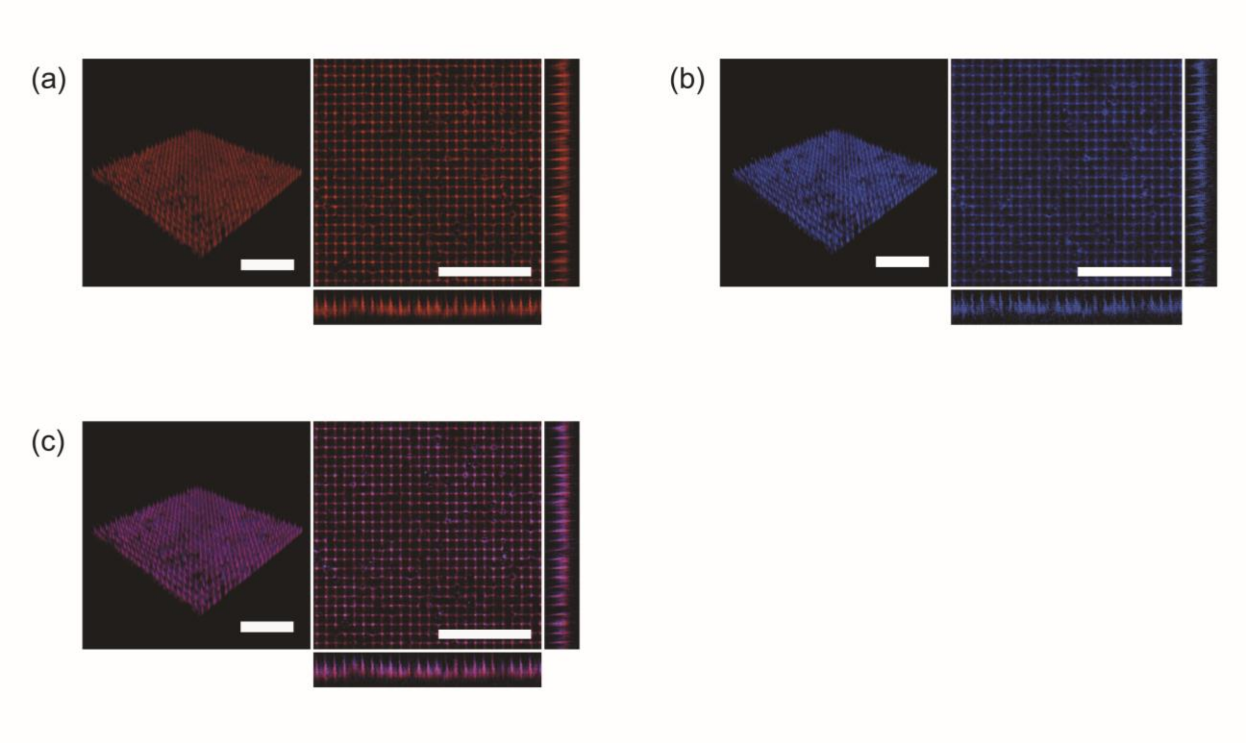

Supplementary figure 1. Confocal microscopy representative image of silicon nanoneedles coated with hyaluronate-chitosan-pDNA polyplex nanofilms. The nanofilm was stained with TAMRA in red **(a)** and pDNA-polyplexes were stained with DAPI **(b)**. Merged image shown in **(c)**. Scale bars represent 20 μm.

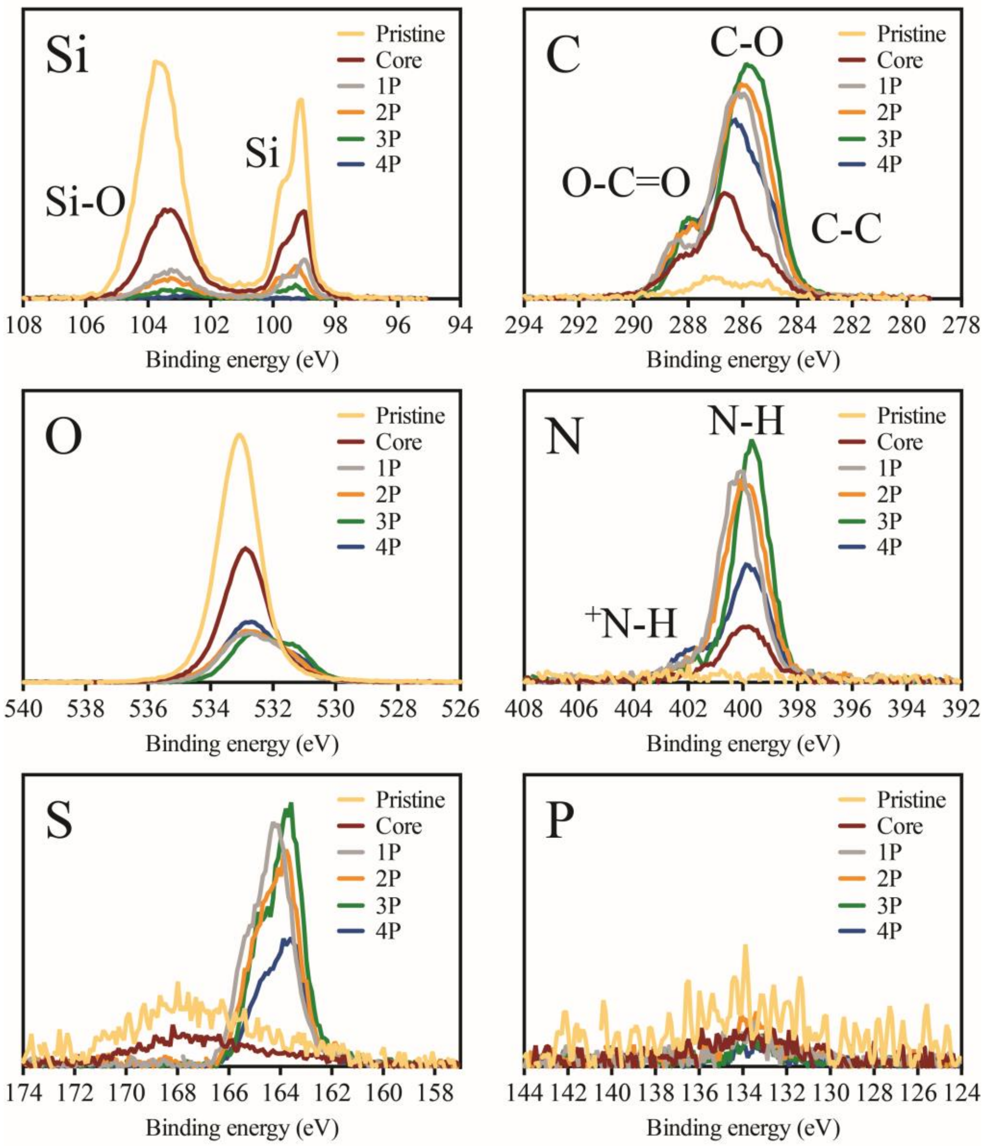

Supplementary figure 2. Carbon, oxygen, nitrogen, sulfur, phosphorus and silicon spectra obtained via XPS from pristine, coated (no polyplex) and nanoneedles coated with 1 to 4 polyplex layers (1P to 4P).

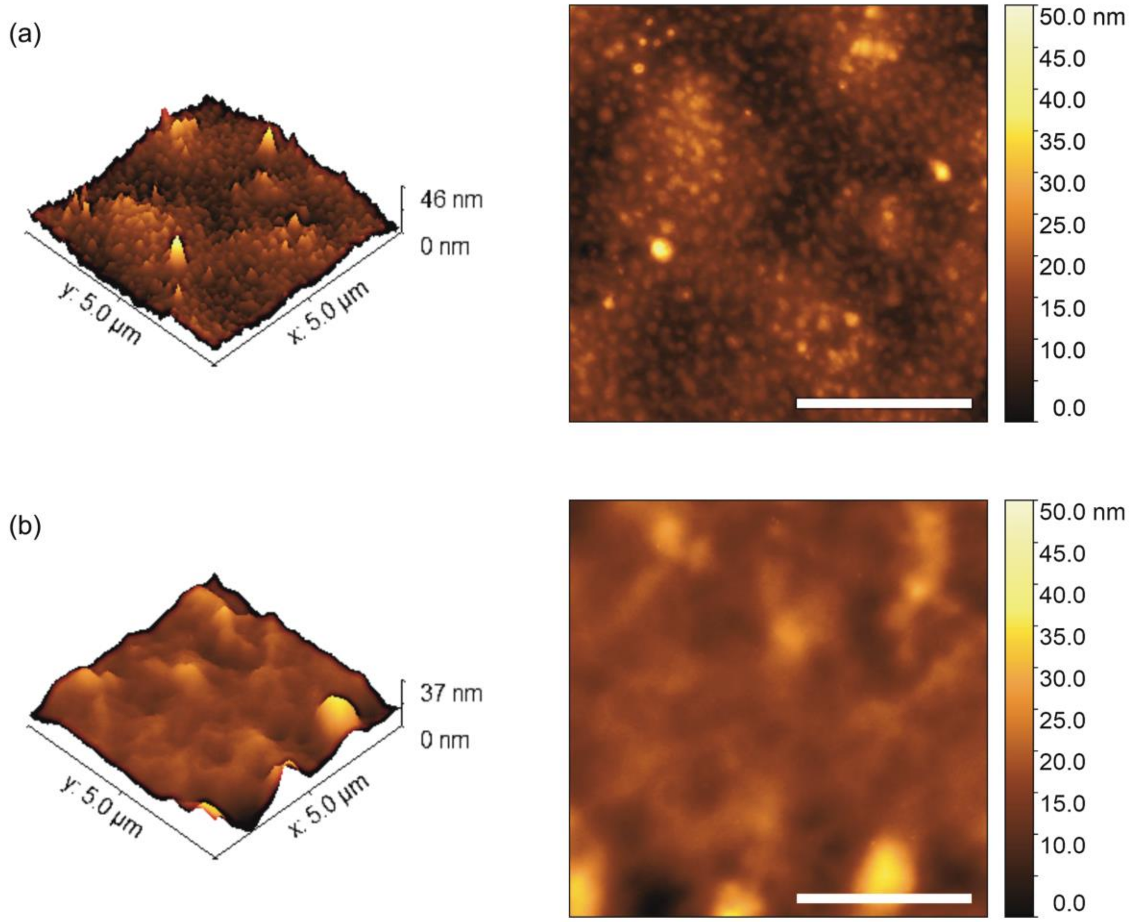

Supplementary figure 3. Surface topography characterization via atomic force microscopy (AFM) for flat silicon substrates coated with **(a)** 4-polyplex nanofilms and **(b)** 5-polyplex nanofilms. Scale bars represent 2 μm.

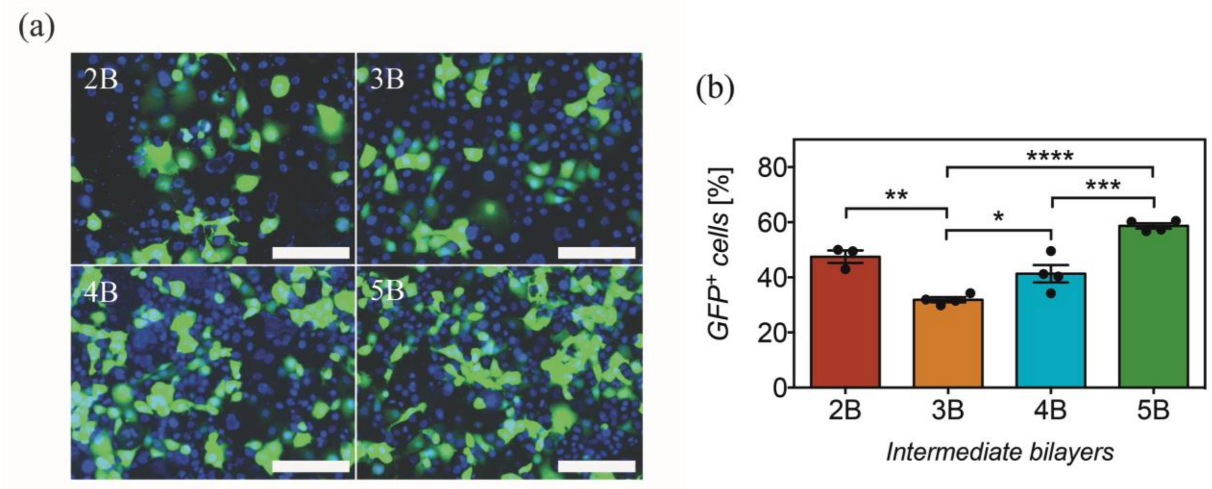

Supplementary figure 4. (a) Fluorescent microscopy images of COS-7 cells transfected and expressing pCAG-GFP plasmid (green) using nanofilms containing 4 polyplex layers with 2 to 5 intermediate bilayers between each polyplex layer (2B to 5B). DAPI (blue) was used as counterstaining. Scale bars represent 100 μm. **(b)** Quantification of the percentage of GPF^{+} cells and via image analysis of COS-7 cells transfected with coated nanoneedles containing 2 to 5 intermediate bilayers (2B to 5B) after 24 hours of incubation. Bars represent the mean ± SEM (N=3). Statistical significance as (*) $p < 0.05$, (**) $p < 0.01$ (***), $p < 0.001$ and (****) $p < 0.0001$, using one-way ANOVA and Sidak's test.

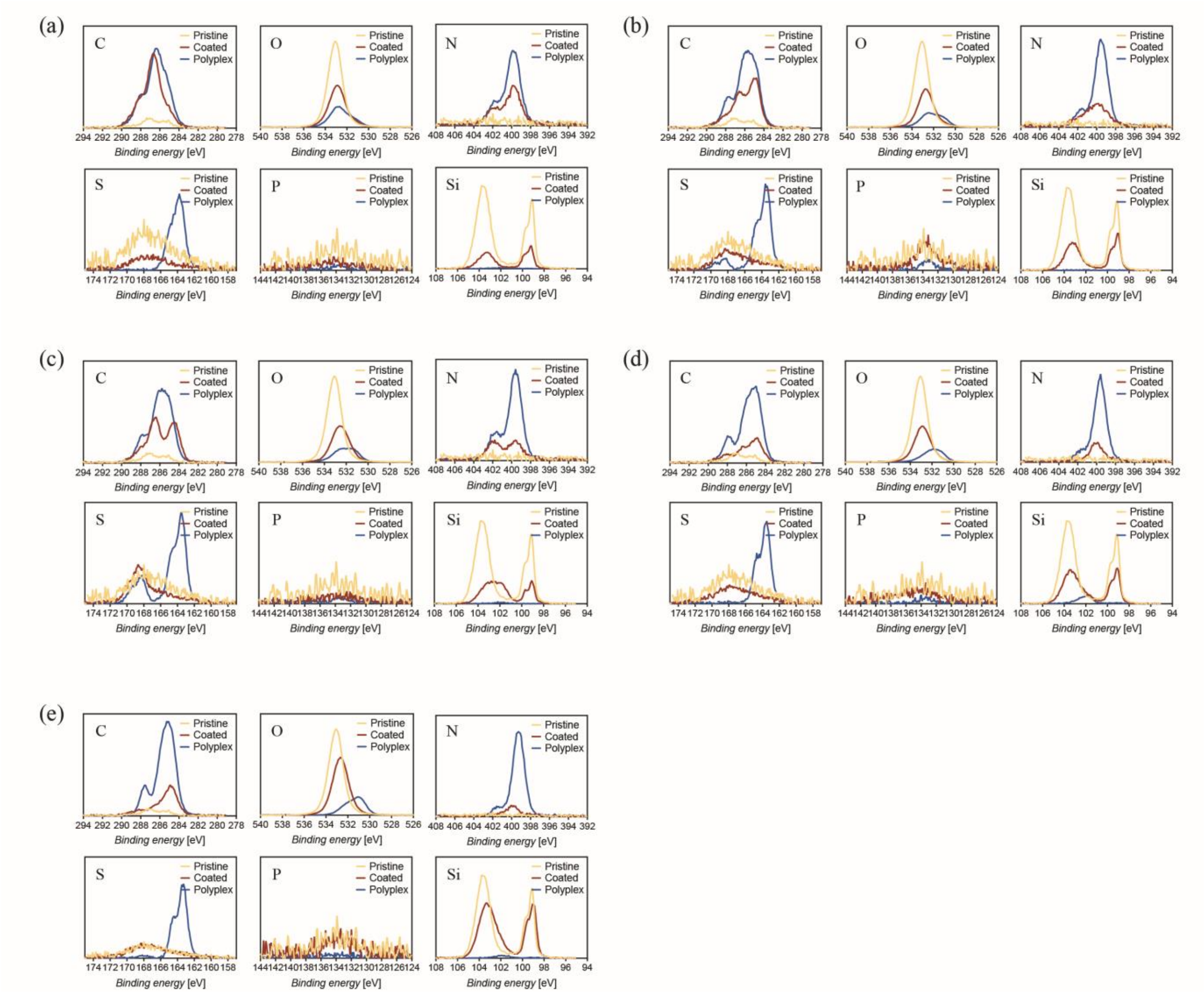

Supplementary figure 5. Carbon, oxygen, nitrogen, sulfur, phosphorus and silicon spectra obtained via XPS from nanoneedles coated with 4 polyplex layers and chitosan - alginate **(a)**, chitosan - chondroitin sulfate **(b)**, chitosan - heparin **(c)**, chitosan - poly(glutamic) acid **(d)** and chitosan - γ poly(glutamic) acid **(e)**.

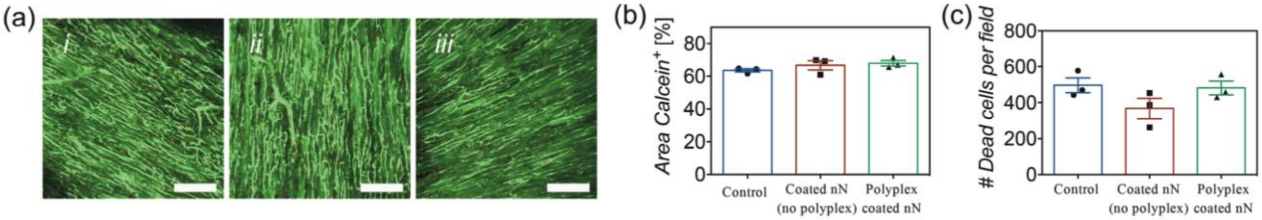

Supplementary figure 6. (a) Confocal microscopy images showing the viability of cardiac slices 24 hours post-transfection using calcein-AM for live cells (green) and ethidium homodimer for dead cells (red). Representative images correspond to control no chip (*i*), coated (no polyplex nanoneedles (*ii*) and polyplex-coated nanoneedles (*iii*). Polyplexes contained pDNA coding for luciferase, to eliminate green fluorescence. Bars represent 200 μm. **(b)** Quantification via image analyses of calcein expression in the slice as area percentage per field and **(c)** number of dead cells per field for control no chip, coated (no polyplex nanoneedles and polyplex-coated nanoneedles. Bars represent the mean ± SEM of three samples (N=3), from an average of three separate images. Differences were not statistically significant, using one-way ANOVA ($p < 0.05$).

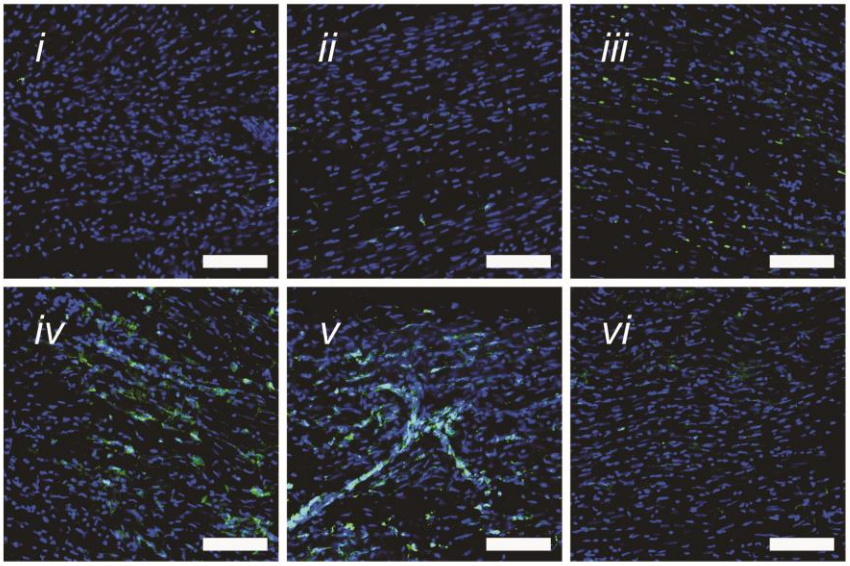

Supplementary figure 7. Confocal microscopy images of cardiac slices at 24 hours of transfection and immunolabelled with anti-GFP (green) and DAPI (blue). Representative images correspond to control no chip (*i*), coated (no polyplex) nanoneedles (*ii*), polyplex-coated flat substrates (*iii*) polyplex-coated nanoneedles (*iv*, *v*) and the non-interfacing side of a slice with polyplex-coated nanoneedles (*vi*). Bars represent 100 μm.
